# Identification of tree species based on the fusion of UAV hyperspectral image and LiDAR data in a coniferous and broad-leaved mixed forest in Northeast China

**DOI:** 10.3389/fpls.2022.964769

**Published:** 2022-09-23

**Authors:** Hao Zhong, Wenshu Lin, Haoran Liu, Nan Ma, Kangkang Liu, Rongzhen Cao, Tiantian Wang, Zhengzhao Ren

**Affiliations:** ^1^ College of Engineering and Technology, Northeast Forestry University, Harbin, China; ^2^ Beijing Institute of Surveying and Mapping, Beijing, China

**Keywords:** hyperspectral, LiDAR, UAV, data fusion, tree species, feature optimization, natural forest

## Abstract

Rapid and accurate identification of tree species *via* remote sensing technology has become one of the important means for forest inventory. This paper is to develop an accurate tree species identification framework that integrates unmanned airborne vehicle (UAV)-based hyperspectral image and light detection and ranging (LiDAR) data under the complex condition of natural coniferous and broad-leaved mixed forests. First, the UAV-based hyperspectral image and LiDAR data were obtained from a natural coniferous and broad-leaved mixed forest in the Maoer Mountain area of Northeast China. The preprocessed LiDAR data was segmented using a distance-based point cloud clustering algorithm to obtain the point cloud of individual trees; the hyperspectral image was segmented using the projection outlines of individual tree point clouds to obtain the hyperspectral data of individual trees. Then, different hyperspectral and LiDAR features were extracted, respectively, and the importance of the features was analyzed by a random forest (RF) algorithm in order to select appropriate features for the single-source and multi-source data. Finally, tree species identification in the study area were conducted by using a support vector machine (SVM) algorithm together with hyperspectral features, LiDAR features and fused features, respectively. Results showed that the total accuracy for individual tree segmentation was 84.62%, and the fused features achieved the best accuracy for identification of the tree species (total accuracy = 89.20%), followed by the hyperspectral features (total accuracy = 86.08%) and LiDAR features (total accuracy = 76.42%). The optimal features for tree species identification based on fusion of the hyperspectral and LiDAR data included the vegetation indices that were sensitive to the chlorophyll, anthocyanin and carotene contents in the leaves, the partial components of the transformed independent component analysis (ICA), minimum noise fraction (MNF) and principal component analysis (PCA), and the intensity features of the LiDAR echo, respectively. It was concluded that the framework developed in this study was effective in tree species identification under the complex conditions of natural coniferous and broad-leaved mixed forest and the fusion of UAV-based hyperspectral image and LiDAR data can achieve enhanced accuracy compared the single-source UAV-based remote sensing data.

## 1 Introduction

Tree species information is a prerequisite for undertaking research on the diversity of forest species, which is essential for constructing prediction models for forest ecosystems. Accurate identification of tree species is of great significance for forest resource monitoring, biodiversity assessment, biomass retrieval, and forest carbon sinks (Lu et al., 2019). To date, the traditional field survey of tree species typically relies on setting up sample plots for a manual survey, which has disadvantages such as high field-work intensity, high cost and long cycle time. In contrast, the application of remote sensing technology in forest inventory has the characteristics of high efficiency, short survey time and low cost, and can reflect the dynamic changes of surface vegetation. With the continuous development of space technology and communication sensing technology, remote sensing has developed from the traditional optical remote sensing stage to multi-source remote sensing *via* different platforms, especially hyperspectral remote sensing and high-precision light detection and ranging (LiDAR) technologies (Feng et al., 2020).

Hyperspectral remote sensing, as a passive remote sensing technology, obtains continuous spectral information by acquiring the electromagnetic waves reflected by ground objects. Compared with other remote sensing technologies, it has the advantages of high spectral resolution and a powerful ability to distinguish nuances of ground objects (Li et al., 2019). Previous studies have demonstrated that hyperspectral technology can be used to identify tree species (Jensen et al., 2012; Fricker et al., 2019; Modzelewska et al., 2020; Wan et al., 2020; Zhao et al., 2021). Feature extraction is the key step of tree species identification by hyperspectral technology and then the extracted features are used together with classification algorithms to classify the image pixels and realize tree species identification. Jensen et al. (2012) identified temperate tree species in urban area using airborne hyperspectral data, and the results showed that the classification accuracy increased from 82% to 91.4% after combining vegetation indices, band means and band ratios with principal component analysis (PCA) transform features compared with PCA method only. Fricker et al. (2019) used airborne hyperspectral images and RGB images for identification of tree species in mixed coniferous forests, and carried out individual tree level studies on the dominant species and dead trees with the aid of convolutional neural networks. Their results showed that the identification accuracy of tree species *via* the hyperspectral image was superior to that of the RGB image. Modzelewska et al. (2020) classified seven different tree species by acquiring airborne-derived hyperspectral images of natural and planted forests. An MNF (minimum noise fraction) transformation was applied to obtain the uncorrelated components from the hyperspectral data and then a support vector machine (SVM) model was used to produce thematic maps of tree species. Their results showed that the overall classification accuracy of planted forests (77%) was higher than that of natural forests (64%). Wan et al. (2020) used GF-5, Hyperion and Landsat8 satellite-derived hyperspectral data to classify mangrove tree species by random forest (RF) and SVM models and the corresponding identification accuracies were 87.12%, 86.82% and 73.89%, respectively. Zhao et al. (2021) extracted spectral features, texture features, vegetation indices and statistical features for feature selection and identification of tree species from the UAV-based hyperspectral images of a protected plantation forest with simple structure in Xinjiang, China and a higher classification accuracy was obtained. To sum up, the accuracy of individual tree species identification in most studies was not very high by using the spectral information of hyperspectral data only. Since hyperspectral data only contains two-dimensional information of the object being measured, which has poor segmentation ability for individual trees, especially under complex forest conditions, most studies on tree species identification *via* airborne and spaceborne hyperspectral images were performed at the plot scale (Wu and Zhang, 2020). Other technologies should be used in combination with hyperspectral technology in order to carry out fine identification at the individual tree level.

The LiDAR is an active remote sensing technology that uses laser light emitted from an optoelectronic sensing device to determine the distance to a target and obtain spatial information about the target (Wang et al., 2019). Compared with traditional optical passive remote sensing, LiDAR data can accurately extract vertical information of forest stands, and this capability offers unparalleled advantages in forestry applications (Man et al., 2020). The general process of tree species identification based on LiDAR data includes individual tree segmentation, features extraction (e.g., three-dimensional texture, clustering degree, structure and echo intensity), and tree species classification (Li et al., 2013; Zhang and Liu, 2013). Sooyoung et al. (2009) used multi-temporal airborne LiDAR data for the identification of tree species at the individual tree level based on the echo intensity of trees before and after defoliation, and the comparison results showed that the LiDAR data after defoliation was more suitable for identification than that before defoliation. Shi et al. (2018a) performed feature extraction and tree species identification at the individual tree level based on LiDAR data acquired by an airborne Riegl LMS-Q680i scanner in a mixed forest in Central Europe. The results showed that the feature of echo intensity provided a higher identification capability compared with the geometric features. Even though the identification of tree species at the individual tree level can be achieved with LiDAR data, a limited number of such studies using LiDAR data only were conducted. The reason for this is that the LiDAR technique lacks information at the spectral dimension level, thus only the geometric and echo features can be used to conduct tree species identification. Clearly, the lack of feature information had certain negative impacts on the accuracy of identification.

It is thus difficult for a single remote sensing data source to meet the high-precision requirements for tree species identification. However, LiDAR and hyperspectral data are highly complementary; therefore, the fusion of the two types of data has been gradually applied in tree species identification. The main idea of combining LiDAR and hyperspectral data for tree species identification is as follows: the LiDAR data is used for individual tree segmentation, and the hyperspectral features and LiDAR features are extracted separately and used together with classification algorithms for tree species identification (Liu et al., 2013; Shen and Cao, 2017). In 2012, Dalponte et al. conducted an identification study based on the acquired airborne LiDAR, multispectral and hyperspectral data. They found that the identification of the tree species was more accurate with the addition of tree height information and the accuracy of identification for the fused LiDAR and hyperspectral data was superior to that of either using just the LiDAR or multispectral data. In 2013, the Chinese Academy of Forestry developed the LiCHy (LiDAR, CCD and Hyperspectral) airborne observation system to obtain the vertical structure, horizontal structure and spectral attributes of ground objects at a higher spatial resolution, which has been widely used in forest resource surveys (Li et al., 2016; Pang et al., 2016; Wu et al., 2018; Jia et al., 2020; Zhang et al., 2020; Pang et al., 2021).

In recent years, the combined UAV-based hyperspectral and LiDAR data have been used for tree species identification in consideration of the flexibility and cost (Sothe et al., 2019; de Almeida et al., 2021; Jiang et al., 2021). For example Sankey et al. (2017, 2018) employed UAV hyperspectral and LiDAR data for monitoring forests with varying tree cover (22%-55%) and densities (1-3.5 trees/10-m cell) and sparse vegetation in arid and semi-arid areas, and used decision tree methods for the identification of tree species. Their results showed that the identification performance was better for the fused data set compared with that of the single data set. Cao et al. (2021) extracted feature information of mangroves in southern China based on UAV-based hyperspectral and LiDAR data, and compared the classification accuracies of three different classifiers (random forest, logic model tree, rotation forest ensemble learning algorithm). The results proved that the addition of the canopy height information from LiDAR could improve the accuracy of tree species identification compared with hyperspectral data alone and the rotation forest ensemble learning algorithm was more accurate and stable in classifying mangrove species. Hartling et al. (2021) used UAV multispectral, hyperspectral, and LiDAR data to conduct a comparative study on tree species identification. The results showed that the identification using the hyperspectral data was significantly better than that of the multispectral data, and the height and shape profile extracted from the LiDAR data were conductive to identifying tree species. Since the research on the fusion of UAV-based hyperspectral and LiDAR data for tree species identification is still in the initial stage, the number of relevant studies is quite limited and most of them focused on simple forest conditions. The studies on tree species identification based on the fusion of UAV-based hyperspectral data and LiDAR data in dense and structurally complex forests such as conifer and broad-leaved mixed forests were rarely reported. It is also known that the hyperspectral image and Lidar data contain huge amount of information, thus the extraction of efficient features is the key step of realizing rapid and accurate tree species identification. However, the best feature combination for conifer and broad-leaved mixed forests was still unknown, which hampered the application of the fused UAV-based hyperspectral data and LiDAR data in different forest conditions. Therefore, it is necessary to conduct individual tree-level species identification based on the UAV-based hyperspectral image and Lidar data on conifer and broad-leaved mixed forests.

The general objective of this study is to develop an accurate tree species identification framework that integrates UAV hyperspectral image and LiDAR data under the complex condition of natural coniferous and broad-leaved mixed forest. Specifically, the objectives are to: (1) obtain the high-precise hyperspectral data and LiDAR point cloud at the individual tree level for a natural coniferous and broad-leaved mixed forest; (2) extract hyperspectral features and LiDAR features, and analyze the feature importance by RF algorithm in order to select appropriate features for the single-source and multi-source data, and (3) perform tree species classification by SVM algorithm and determine the best feature combination for tree species identification in the study area.

## 2 Study area and data acquisition

### 2.1 Overview of the study area

**Figure 1:**
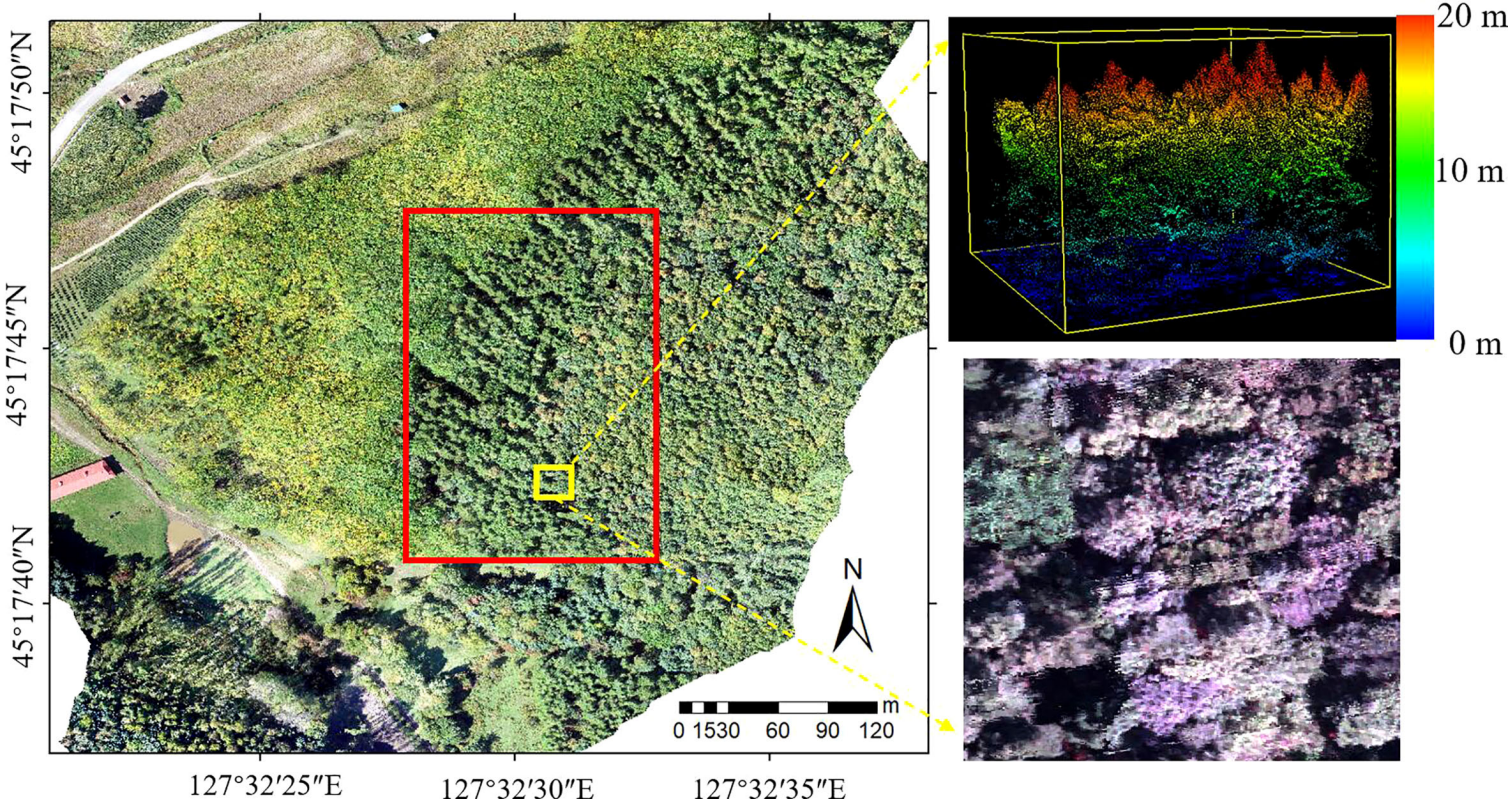
Location of the study area (The red frame of the RGB image on the left is the study area; LiDAR and hyperspectral zoomed-in views are on the right).

The study area (**Figure 1**) is located in Maoershan Experimental Forest Farm in Shangzhi City, Heilongjiang Province of China (127°30′~127°34′ E, 45°20′~45°25′ N), which is a part of the western slope region of Zhangguangcai Range in the Changbai Mountains. The area is a low hilly area with an average slope of 10° and an average elevation of 300 m. The region has a temperate continental monsoon climate, with an average annual temperature and precipitation of 3.1°C and 629 mm, respectively. The soil is fertile, and the soil types are mainly dark brown soil, white mud soil, meadow soil, swamp soil, etc. The flora belongs to the Changbai Mountain flora, and the existing stand types include natural secondary forests at different stages after the destruction and succession of the original zonal climax community. Major arbor species include *Populus davidiana*, *Ulmus pumila*, *Betula platyphylla*, *Fraxinus mandshurica*, *Phellodendron amurense*, *Juglans mandshurica, Quercus mongolica*, *Acer pictum*, *Tilia amurensis*, *Pinus koraiensis* and *Larix gmelinii.*

### 2.2 Data acquisition

#### 2.2.1 UAV data acquisition

Before the flight of the UAV, the flying path for the study area was determined. The flight was conducted on August 26, 2021 and the weather was clear and cloudless, and the wind speed was less than 3.0 m/s. The LiDAR sensor was mounted on an UAV Pegasus D200 (Feima Robotics Technology Company), the flight speed was set to 5.0 m/s, and the flight altitude was 80 m. The laser source was a RIEGL mini VUX-1UAV. A measurement distance of >250 m was employed with an accuracy range of ±1 cm. The number of echoes was 5, the echo intensity was 16 bit, the wavelength was 905 nm, and the point density was about 180 points/m^2^.

The hyperspectral imaging sensor was mounted on an UAV DJI M300RTK, with a flight speed of 4.5 m/s and a flight height of 100 m. The hyperspectral imaging sensor was a Resonon Pika L. The wavelength range was 400–1000 nm, the spectral resolution was 2.1 nm, and the pixel size was 5.86 µm. The shooting method was linear push-broom imaging, and the spatial resolution of the hyperspectral images was 10 cm.

#### 2.2.2 Ground survey data

On September, 2021, the tree species in the sample plot were investigated in detail. The RGB image obtained by the UAV for the sample plot was acquired and printed. Then, the actual investigation of different tree species was carried out in the sample plot, and the tree species and locations were marked on the drawing of the RGB image. Combined with a visual interpretation method, the detailed distribution information of tree species in the study area was obtained.

## 3 Methods

**Figure 2:**
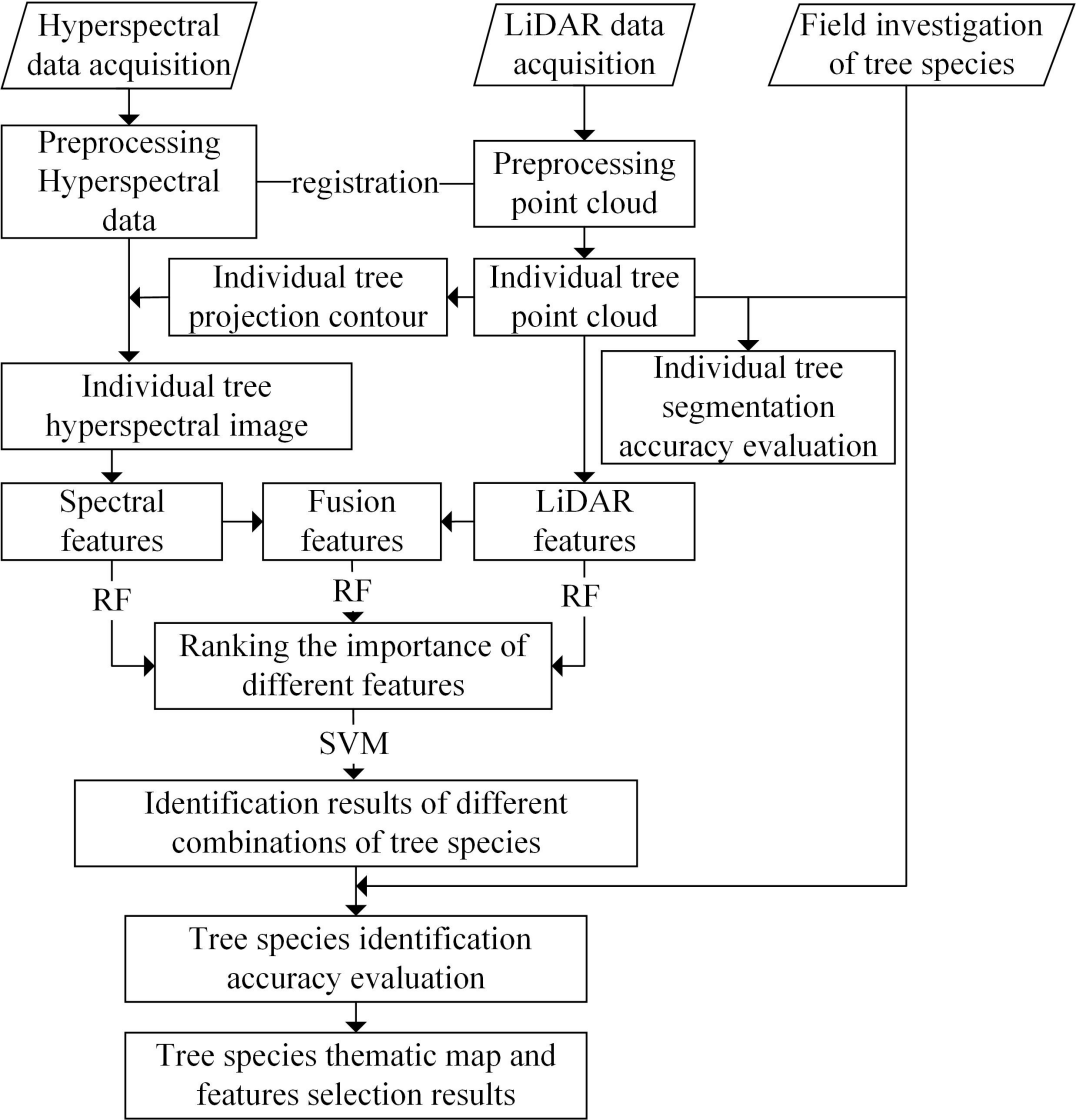
Flowchart for the identification of tree species based on fusion of the hyperspectral and LiDAR data.

At first, the point clouds of individual trees were obtained by using the distance-based point cloud clustering algorithm for segmentation of individual trees based on the LiDAR data, and the UAV hyperspectral image was segmented by the point cloud projection outline to obtain the hyperspectral data of the individual trees. We then calculated and extracted the point cloud features and the hyperspectral features for the individual trees. After fusing the two types of features, the average value reduction algorithm of the Gini coefficient in the RF algorithm was used to calculate the importance of the features; the SVM algorithm was also used to complete the identification of the tree species. Next, the feature screening results were obtained according to the results for the accuracy of identification of the tree species. Finally, a thematic map for the tree species was produced. The flowchart of the overall process is presented in **Figure 2**.

### 3.1 Data preprocessing

The original point cloud data of the UAV LiDAR was denoised by LiDAR360 software to remove the high-level gross errors caused by flying objects (such as birds), and the low-level gross errors caused by multipath errors or laser rangefinder errors during measurement. Then, an improved progressive TIN densification proposed by (Zhao et al., 2016) was used to separate the ground points, and the parameters were selected as follows: moderate terrain scene, iteration angle 10°, and iteration distance 1.5m. Finally, the point cloud data were normalized according to the separated ground points and cut to obtain the point cloud data for the study area. For the original hyperspectral data, stitching, radiometric calibration, geometric correction, and atmospheric correction were implemented, and then the Savitzky-Golay convolution smoothing algorithm was used to remove the burr noise from the hyperspectral image. During data collection, the GNSS and IMU carried by the UAV can ensure the spatial accuracy of the data. However, there are still slight deviations between the two types of data. In order to improve the accuracy of hyperspectral data, the coordinates of the common ground objects such as the boundary points of tree crowns, road corners were extracted from point cloud data, which were used together with the function of quadratic polynomial correction in ArcGIS to realize the registration of hyperspectral data. The data error was within 1 pixel (10 cm), which can fully satisfy the requirements of this experiment.

### 3.2 Data acquisition for individual trees

The acquisition of accurate data for individual trees laid the foundation for the identification of tree species at the individual tree level. The three-dimensional information contained in the LiDAR data ensures the unparalleled advantages in individual tree segmentation compared with other remote sensing data. Generally, two basic approaches are available for the segmentation of individual tree point clouds. The first method is based on canopy height model (CHM), which is to compress the three-dimensional point cloud data to a two-dimensional plane, thus reducing the computational complexity; however, this data reduction approach causes the loss of information, and the point cloud difference will produce errors in the process of CHM generation, resulting in a relatively low segmentation accuracy for individual trees under a complex stand with a high canopy density (de Almeida et al., 2021). The other method is to use segmentation of individual trees which directly faces the point cloud. This method can not only use more point cloud spatial information to improve the accuracy of segmentation (Li et al., 2020), but also can be used to extract structural parameters and features of the individual tree based on the point cloud data. Therefore, this study used a distance-based point cloud clustering algorithm (Li et al., 2012) to segment the forest stand point cloud data and to obtain the point cloud data at the individual tree level.

The hyperspectral data for individual trees were obtained based on the LiDAR point cloud data of individual trees. Given that the LiDAR data was registered with the hyperspectral data, the positions of the individual trees in LiDAR data corresponded to those in the hyperspectral data. The concave hull algorithm was used on the point cloud data of the individual tree to obtain the projection outline vector file, and then the projection profile was used to segment the registered hyperspectral image to obtain the hyperspectral canopy data.

### 3.3 Feature extraction

#### 3.3.1 Hyperspectral feature extraction

Hyperspectral data contains a massive amount of spectral information, which may be used for the accurate identification of tree species. Although the complete hyperspectral image for individual trees has been obtained through segmentation, the overlapping and crossing of tree branches at the edges of the hyperspectral image may result in the existence of mixed pixels. In addition, previous studies have demonstrated that the spectral signal of the tree canopy illuminated by sunlight was dominated by first-order scattering, which was less affected by soil and shade, hence the data set was more suitable for tree crown modeling and identification (Coops et al., 2003). Therefore, in order to obtain more accurate hyperspectral information at the individual tree level, this study selected the spectral average of 100 sunlight pixels around the center of the individual tree as the hyperspectral data of individual tree. Since the difference in reflectivity between sunlight pixels and shadow pixels in the near-infrared region was obvious (Shen and Cao, 2017),this study determined the sunlight pixels at the 850 nm near-infrared band with reflectivity greater than 0.25. The hyperspectral data obtained at the individual tree level were subjected to PCA in order to select the first 10 components (PCA1~PCA10), and minimum noise fraction rotation (MNF) to select the first 15 components (MNF1 ~MNF15), and independent component analysis (ICA) to select the first 20 components (ICA1~ICA20), respectively. In addition, 18 vegetation indices were extracted, as shown in **Table 1**. So a total of 363 hyperspectral features including 300 original bands (bands 1~300) and 63 components and indices were selected for identification purposes.

**Table 1:** Calculation table for the vegetation index.

Property	Vegetation Index	Description	Computing method
Broadband greenness	NDVI	Normalized difference vegetation index	(ρ865 - ρ672)/(ρ865 + ρ672)
	SRI	Simple ratio index	ρ865/ρ672
	EVI	Enhanced vegetation index	2.5 × ((ρ865 - ρ672)/(ρ865 + 6 × ρ672 -7.5 × ρ464 + 1))
	ARVI	Atmospherically resistant vegetation index	(ρ865 - (2 × ρ672 - ρ464))/(ρ865 + (2 × ρ672 - ρ464))
	SGI	Sum green index	Average value:500 – 599nm
Narrowband greenness	RENDVI	Red edge normalized difference vegetation index	(ρ750 - ρ705)/(ρ750 + ρ705)
	MRESRI	Modified red edge simple ratio index	(ρ750 - ρ445)/(ρ705 + ρ445)
	MRENDVI	Modified red edge normalized difference vegetation index	(ρ750 - ρ705)/(ρ750 + ρ705 – 2 × ρ445))
	VREI1	Vogelmann red edge index 1	ρ740/ρ720
	REPI	Red edge position index	Max first derivative: 690 – 740 nm
Light use efficiency	PRI	Photochemical reflectance index	(ρ570 - ρ531)/(ρ531 + ρ570))
	SIPI	Structure insensitive pigment index	(ρ800 - ρ445)/(ρ800 - ρ680)
	RGRI	Red green ratio index	$\sum_{\text{i}=600}^{699}\rho_{i}/\sum_{\text{i}=500}^{599}\rho_{i}$
Leaf pigments	CRI1	Carotenoid reflectance index 1	(1/ρ510) - (1/ρ550)
	CRI2	Carotenoid reflectance index 2	(1/ρ510) - (1/ρ700)
	ARI1	Anthocyanin reflectance index 1	(1/ρ550) - (1/ρ700)
	ARI2	Anthocyanin reflectance index 2	ρ800[(1/ρ550) - (1/ρ700)]
Canopy water content	WBI	Water band index	ρ970/ρ900

#### 3.3.2 LiDAR feature extraction

The LiDAR point cloud data contains not only accurate 3D information of the target, but also information on the reflection intensity, thus the technique provides a strong capability for accurate segmentation of individual trees, and this information clearly aids the tree species identification process. Based on the information of the point cloud for individual trees, four parameters, namely, tree height (*H_T_*), crown width (*W_C_*), crown area (*A_C_*) and crown volume (*V_C_*), were extracted in the LiDAR360 software, and three shape features associated with individual trees, that is, the ratio of the crown width to tree height (*R*_W/H_), the ratio of the crown area to tree height (*R*_A/H_) and the ratio of the crown volume to tree height (*R*_V/H_) were computed according to the above parameters. The formulae used for the calculations are given in **Table 2**.

**Table 2:** Calculation of shape features.

Features	Calculation formulae
*R*_W/H_	*R*_W/H_ = *W_C/_H_T_*
*R*_A/H_	*R*_A/H_ = *A_C/_H_T_*
*R*_V/H_	*R*_V/H_ = *V_C/_H_T_*

The clustering degree of the point cloud for different tree species is different. Therefore, the average height (H_n_mean), the standard deviation (H_n_std), the coefficient of variation (H_n_CV), the skewness (H_n_S), the kurtosis (H_n_K) of the point cloud of individual trees and the cumulative heights of 25%, 50%, 75% and 95% (H_n_25, H_n_50, H_n_75, H_n_95) for the point cloud of individual trees were calculated, respectively. Among them, n = 0, 1, 2, which represents the total echo point cloud, the first echo point cloud and the second echo point cloud, respectively. Differences in the morphological structure of different tree species may also lead to some differences in the spatial distribution of the respective point clouds, thus the number of points at different quantile heights can be used as a reflection of the tree structure (Lu et al., 2019). Consequently, the ratio (H_n_P_m_) of the number of point clouds in the height range of 0-20%, 20-40%, 40-60%, 60-80%, and 80-100% to the total number of point clouds for individual trees were extracted as distribution features of the point clouds. The calculation is expressed as Eq. (1), where n = 0, 1, 2, representing the total echo point cloud, the first echo point cloud and the second echo point cloud, respectively; *P*_m_ is the proportions of point clouds within a height range, where m= 0-20%, 20-40%, 40-60%, 60-80%, and 80-100%; $N_{P_{m}}^{n}$ is the number of different echo point clouds within the height range; $N^{n}$ is the total number of echo point clouds for an individual tree.


(1) $$H_{n}P_{m}=\frac{N_{P_{m}}^{n}}{N^{n}}$$

Since the intensity features of the echo had high degree of importance for identification of tree species (Shi et al., 2018a), the average intensity (I_n_mean), the standard deviation (I_n_std), the coefficient of variation (I_n_CV), the skewness (I_n_S), the kurtosis (I_n_K) of the point cloud for individual trees were calculated, respectively. Among them, n = 0, 1, 2, which represents the total echo point cloud, the first echo point cloud and the second echo point cloud, respectively. As a result of the above extraction and calculation, the total number of point cloud features for LiDAR in this study was 60.

### 3.4 Ranking of feature importance

A total of 423 hyperspectral features and LiDAR point cloud features were obtained by the above methods. If all the features were involved in recognition of the tree species, this would increase the computational complexity and workload of the recognition process, and the high-dimensional features would reduce the accuracy of the tree species identification due to the existence of the Hughes phenomenon (Luo et al., 2005; Richards and Jia, 2008; Taskin et al., 2017). The RF algorithm has a strong advantage for assessing the importance of variables (Ziegler and Konig, 2014), thus this study used the Gini exponential mean descent method in the RF algorithm to analyze the importance of the features of the hyperspectral image (HSI), LiDAR features, and HSI+LiDAR fusion features, respectively. After obtaining the ranking, correlation analysis was performed on these features, and only the one with the highest importance was retained among the features with high correlation.

### 3.5 Identification of tree species

The SVM algorithm is a supervised machine learning method based on statistical theory and the main idea is to generate a random hyperplane which keeps moving until the samples belonging to different categories are located on both sides of the hyperplane, thus it is a method specifically designed for classifying small sample training areas. The SVM algorithm can remedy the shortcomings of traditional classification methods such as the maximum likelihood method in the case of large volumes of data with high-dimensional and multi-source features and improve the generalization performance, thus it is widely used in the remote sensing field (Bahria et al., 2011; Liu et al., 2013; Zhang and Liu, 2013; Xun and Wang, 2015). This study used the SVM classification algorithm to obtain the identification results for the tree species *via* the different data sources and the different combinations of features by gradually increasing the features according to the importance of the features.

### 3.6 Verification of accuracy and acquisition of optimal features

In order to evaluate the capability of different feature combinations in tree species identification, 60% samples for each species was selected randomly as the modeling sample and the remaining 40% were used as the test samples. The accuracy of the results was assessed using the producer accuracy (PA), the user accuracy (UA), the commission, the omission, the overall accuracy (OA) and the Kappa coefficient. The feature combination with the highest and stable identification accuracy of tree species was taken as the optimal feature screening result of tree species identification in the study area. According to the tree species identification results, a thematic map of tree species in the sample plot was made. Finally, box charts were made for the selected features to analyze the identification ability of different features for tree species.

## 4 Results and analysis

### 4.1 Segmentation results for individual trees

#### 4.1.1 LiDAR point cloud segmentation results for individual trees

**Figure 3:**
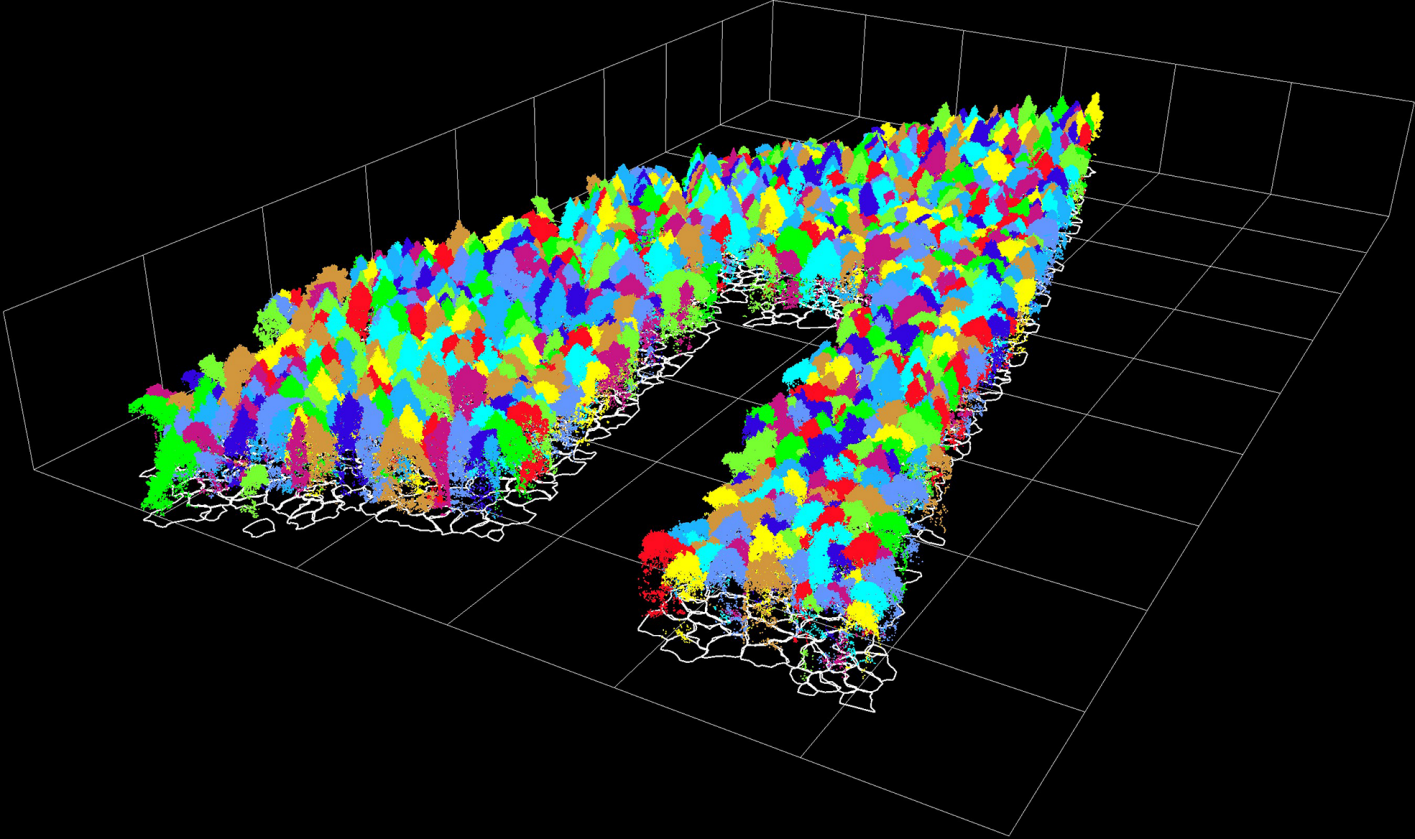
The point cloud and the projection profile of individual trees.

There were 1040 dominant trees in the sample plot, and 936 trees were detected during segmentation, of which 880 were classified correctly. Most of the mis-segmented trees were under-segmented, probably because the sample plot was natural coniferous and broad-leaved mixed forest, with a complex stand structure, high densities and overlapping canopies. A small amount of over-segmentation was found to exist in the tall broadleaf canopy. The rate of detection for individual trees was 90%, and the total accuracy for individual tree segmentation was 84.62%. The correctly segmented individual trees, including 132 *Juglans mandshurica* (JM), 363 *Larix gmelinii* (LG), 223 *Tilia amurensis* (TA), 73 *Quercus mongolica* (QM), and 89 *Ulmus pumila* (UP). The results for the individual trees segmentation and the projection profile are illustrated in **Figure 3**.

#### 4.1.2 Segmentation results for hyperspectral image

**Figure 4:**
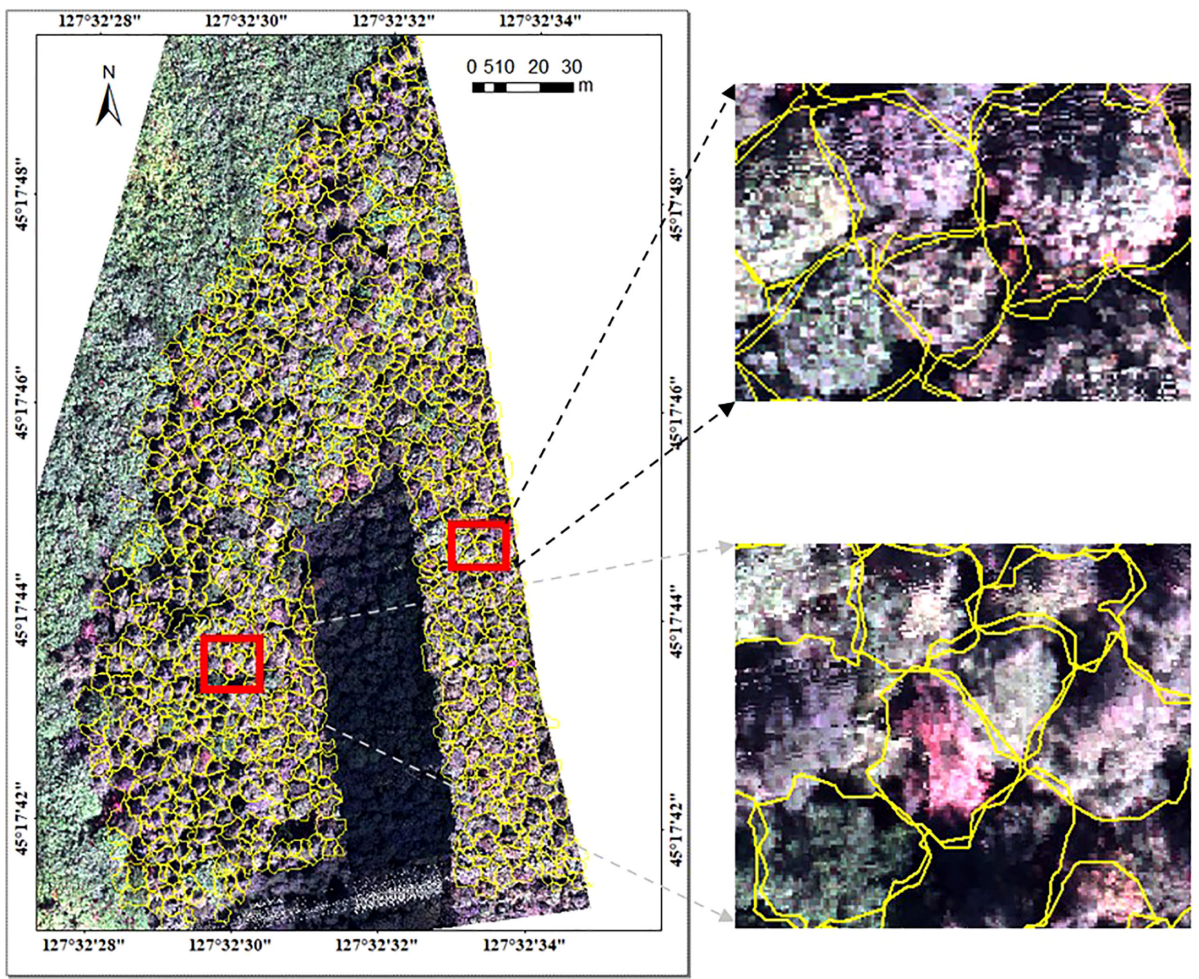
Segmentation results for hyperspectral image.

**Figure 5:**
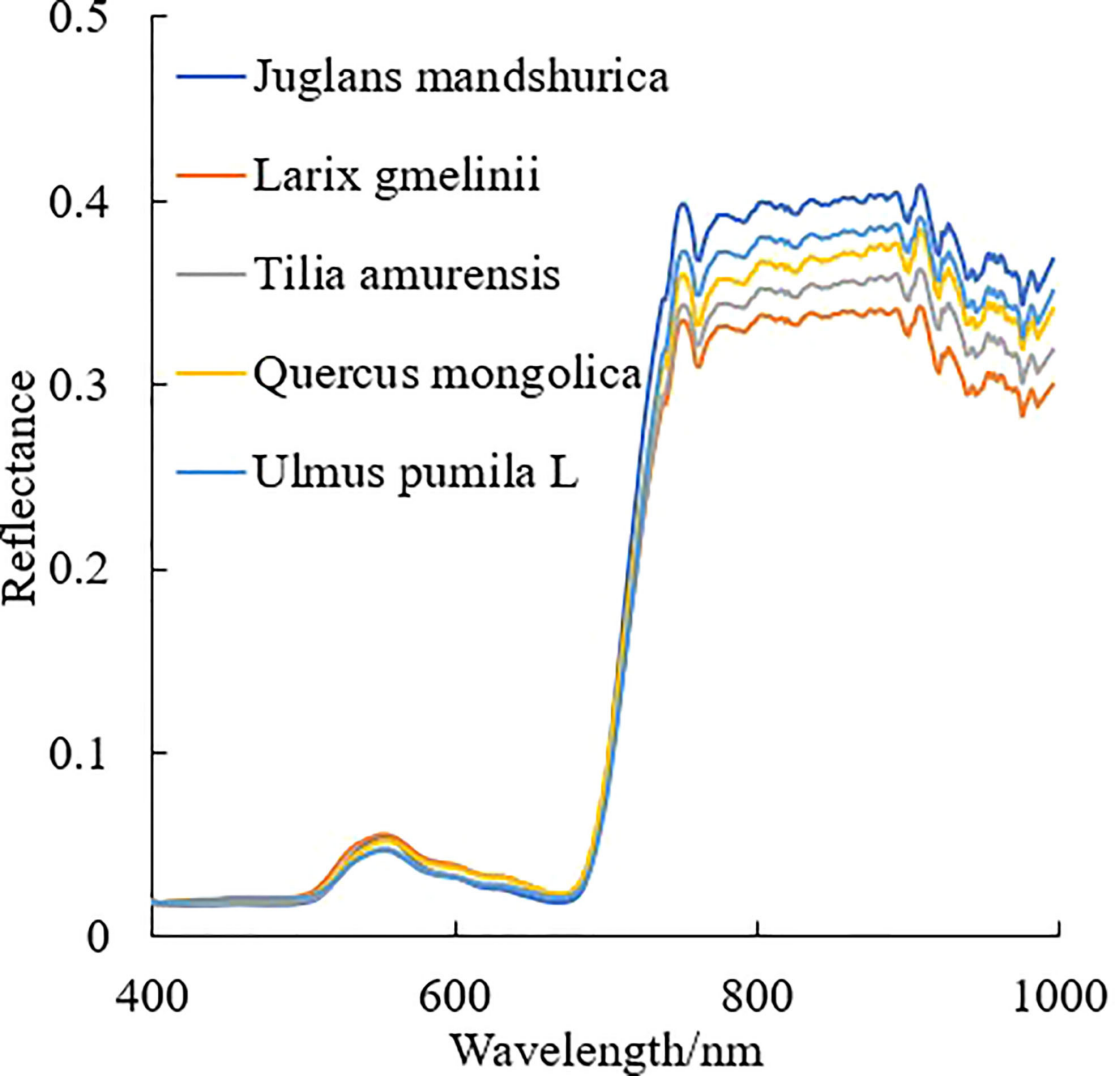
Average spectral curves for different tree species.

The hyperspectral image was segmented based on the point cloud projection profile of individual tree to obtain the hyperspectral data. The segmentation results for the hyperspectral image and the average spectral curve of the central sunlight pixels in the hyperspectral image for different tree species are shown in **Figures 4**, **5**, respectively. Some of the strip data in the acquired hyperspectral images were anomalous due to cloud shadow, which were eliminated for identification purposes. It can be seen from **Figure 4** that the concave hull algorithm can well describe the individual tree canopy, and the central area of the canopy corresponds accurately. Some of the tree canopy borders in the hyperspectral image are dark in color, which is not land but low shadows at the edge of the tree canopy. It can be seen from **Figure 5** that the average spectral curve corresponding to the central sunlight pixels of the hyperspectral image for different tree species shows the differences in reflectance in the visible light of green light bands, and the differences are more significant in the near-infrared bands.

### 4.2 Results of feature extraction and ranking importance

**Figure 6:**
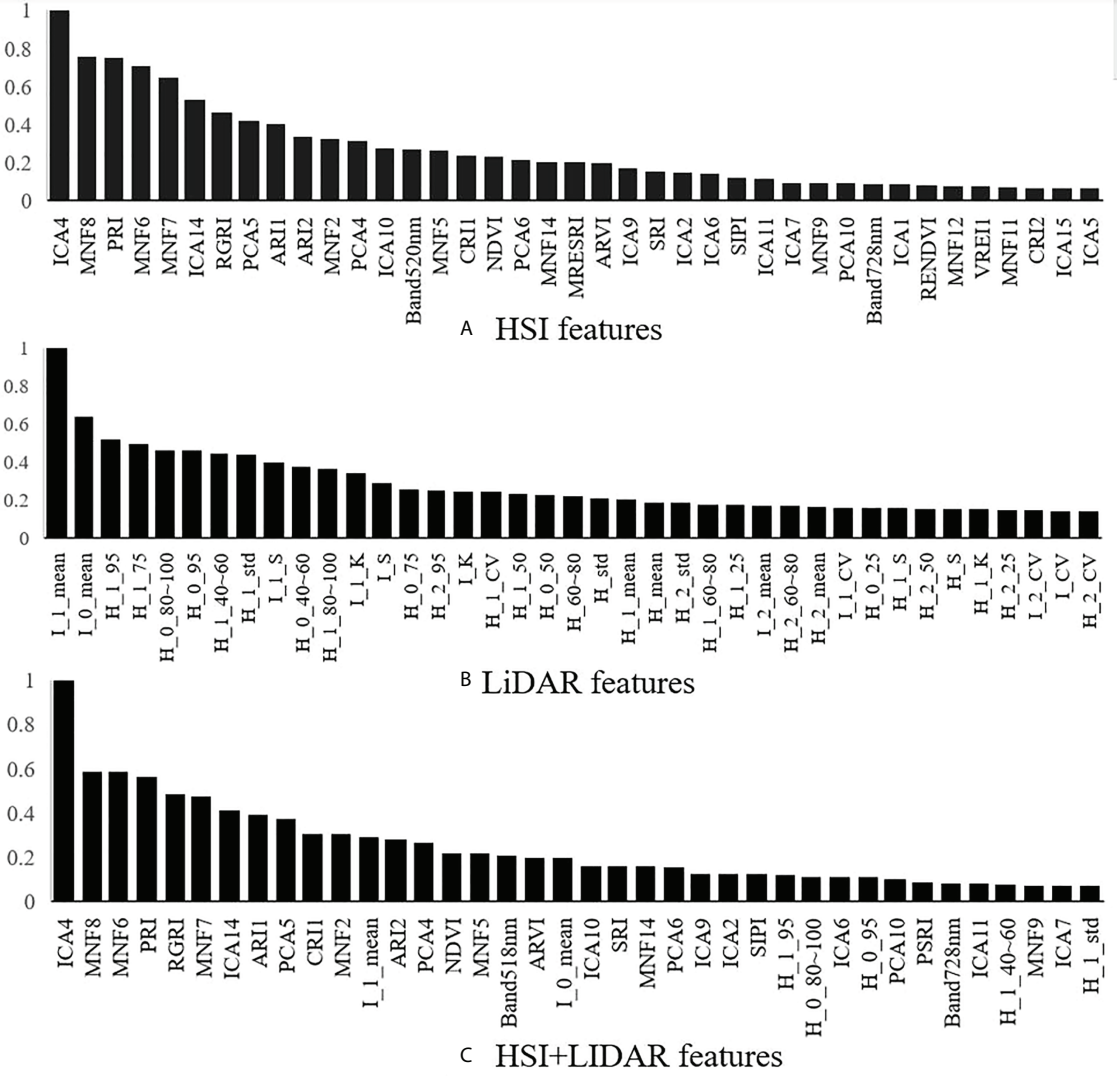
The ranking of features based on importance.

Based on the point cloud and the hyperspectral data of individual trees, the identification features for the tree species were extracted. The top 40 normalized results for each type of features (i.e., HSI features, LiDAR features, and HSI+LiDAR features) in terms of ranking importance are shown in **Figure 6**. With regard to the features extracted from the hyperspectral data, the MNF, the ICA, and the PCA transformed components and the vegetation indices have higher importance compared to the original spectrum. For the features extracted from the LiDAR data, the features of first and total echo intensity are ranked as the top two features. The ranking of the HSI+LiDAR features shows that the importance of the spectral features is generally stronger than that of the LiDAR features, which indicates that the LiDAR data contains less information pertinent to the identification of tree species in comparison with that of the hyperspectral data.

### 4.3 Identification results of tree species

**Figure 7:**
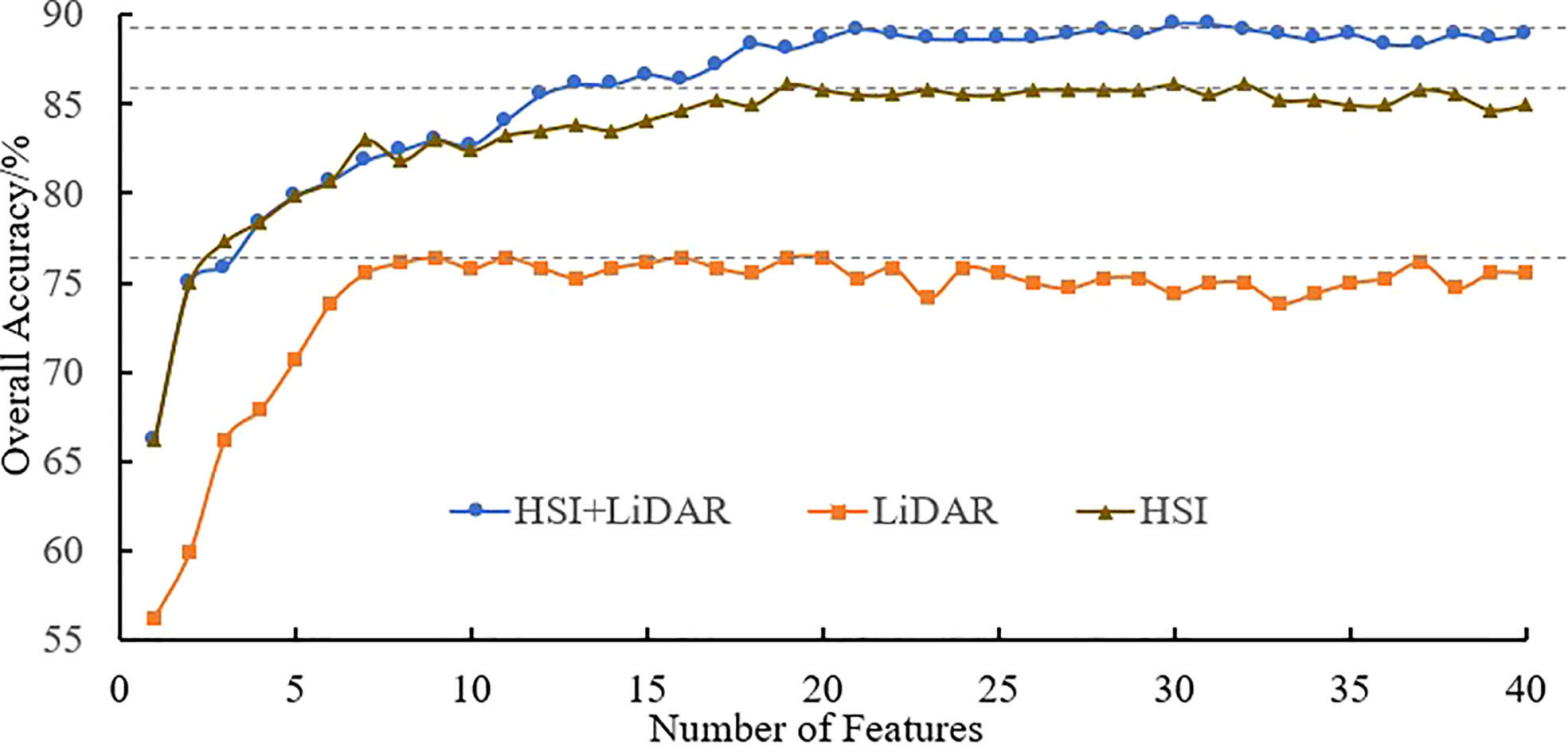
Accuracy of identification of tree species.

Based on the SVM algorithm, the three types of data features, namely, HSI, LiDAR and HSI+LiDAR, were modeled and subjected to the identification process by gradually increasing the number of features from 1 to 40 according to their relative importance. The variation tendency of accuracy based on multiple (120 times) identification results of tree species is shown in **Figure 7**.

It can be seen from **Figure 7** that an increase in the number of features can result in a significant improvement in the accuracy when the number of features is relatively small. However, when the number of features reaches a certain number, the trend in the curves becomes more stable. The optimal accuracy can be realized when the number of HSI+LiDAR features, LiDAR features and HSI features is 21, 9, and 19, respectively. The optimal results of identification and the indices for accuracy evaluation based on the three types of features are presented in **Tables 3**, **4**, and **5**.

**Table 3:** Optimal results of tree species identification and accuracy indices based on HSI features.

Class	JM	LG	TA	QM	UP	User accuracy (%)	Commission (%)
JM	42	4	2	1	2	82.35	17.65
LG	0	139	3	0	2	96.53	3.47
TA	6	1	77	6	5	81.05	18.95
QM	3	0	3	21	3	70.00	30.00
UP	2	1	4	1	24	75.00	25.00
Producer accuracy (%)	79.25	95.86	86.52	72.41	66.67	OA (%)	86.08
Omission (%)	20.75	4.14	13.48	27.59	33.33	Kappa	0.81

**Table 4:** Optimal results of tree species identification and accuracy indices based on LiDAR features.

Class	JM	LG	TA	QM	UP	User accuracy (%)	Commission (%)
JM	37	3	4	2	2	77.08	22.92
LG	3	132	6	0	3	91.67	8.33
TA	10	9	66	8	10	64.08	35.92
QM	1	0	7	17	4	58.62	41.38
UP	2	1	6	2	17	60.71	39.29
Producer accuracy (%)	69.81	91.03	74.16	58.62	47.22	OA (%)	76.42
Omission (%)	30.19	8.97	25.84	41.38	52.78	Kappa	0.67

**Table 5:** Optimal results of tree species identification and accuracy indices based on HSI+LiDAR features.

Class	JM	LG	TA	QM	UP	User accuracy (%)	Commission (%)
JM	43	2	2	0	1	89.58	10.42
LG	0	143	2	0	2	97.28	2.72
TA	5	0	78	4	4	85.71	14.29
QM	2	0	3	24	3	75.00	25.00
UP	3	0	4	1	26	76.47	23.53
Producer accuracy (%)	81.13	98.62	87.64	82.76	72.22	OA (%)	89.20
Omission (%)	18.87	1.38	12.36	17.24	27.78	Kappa	0.85

**Figure 8:**
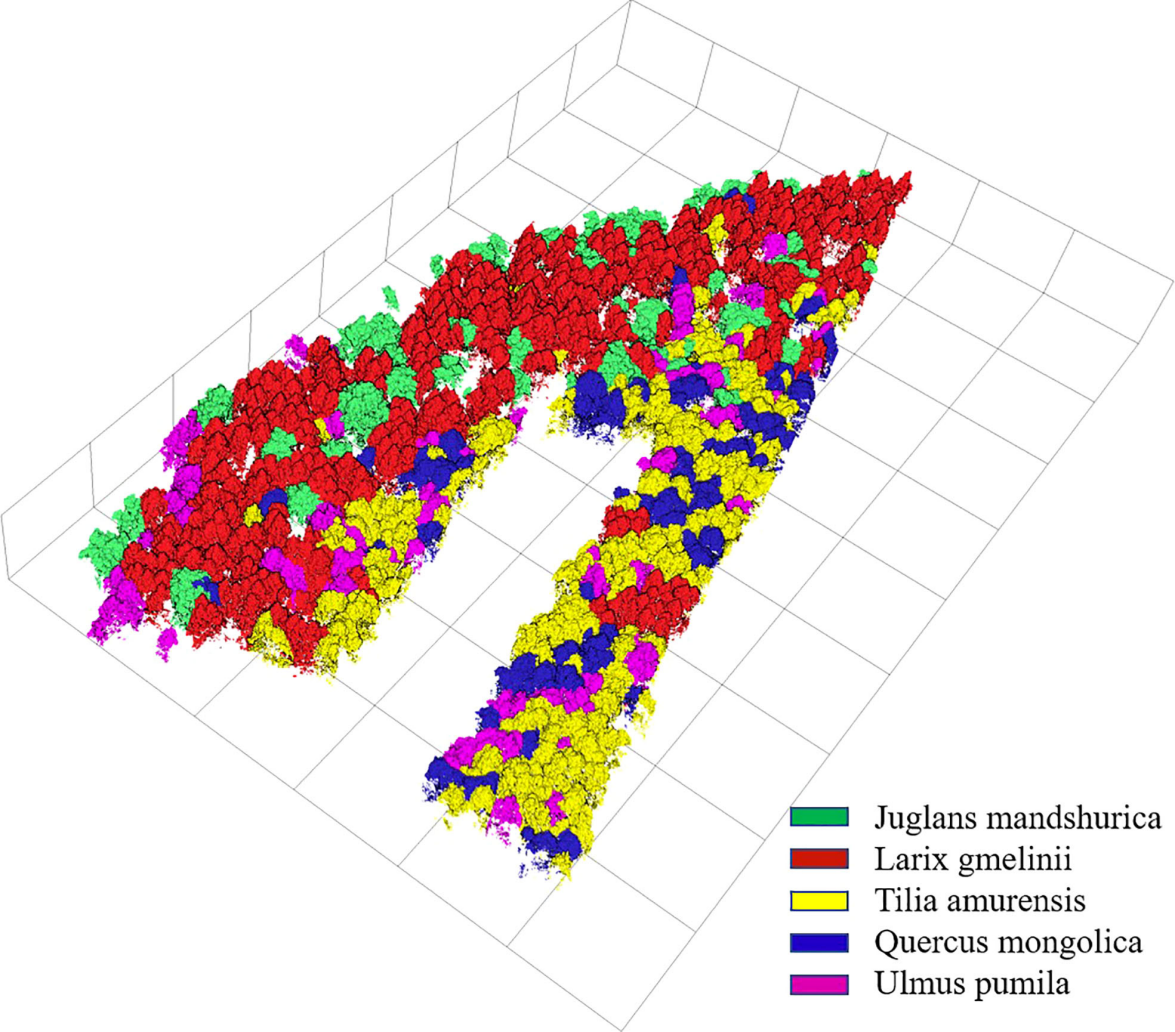
Thematic map of tree species.

The identification results and evaluation indices in **Tables 3**, **4**, and **5** show that the three types of data had better performance for the identification of coniferous and broad-leaved tree species. Although the LiDAR data yielded the poorest identification capability, the fusion of LiDAR with HSI resulted in an enhanced identification performance compared with the use of the HSI data only. In general, the combined HSI+LiDAR features yielded the highest accuracy of identification, followed by the HSI features and LiDAR features. The thematic map of tree species identification based on the optimal results for the HSI+LiDAR feature combination is presented in **Figure 8**.

### 4.4 The capability of features for tree species discrimination

**Figure 9:**
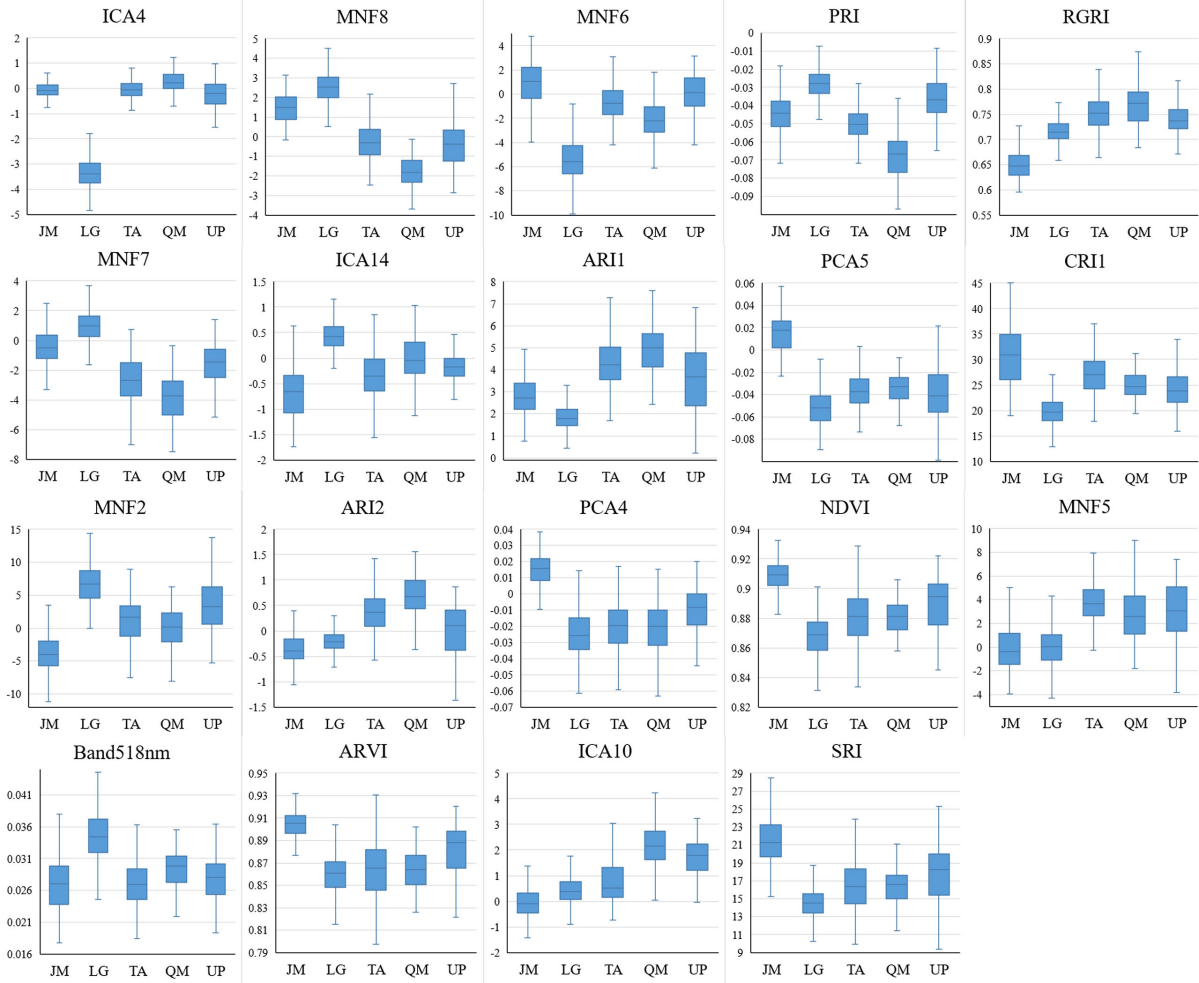
HSI features capability for tree species discrimination (JM, *Juglans mandshurica*; LG, *Larix gmelini*; TA, *Tilia amurensis*; QM, *Quercus mongolica*; UP, *Ulmus pumila*).

**Figure 10:**
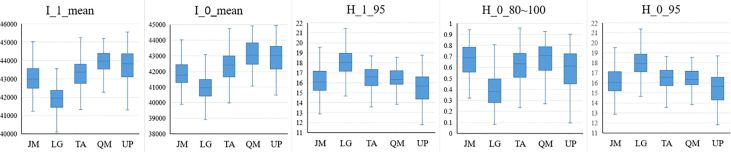
LiDAR features capability for tree species discrimination (JM *Juglans mandshurica*; LG *Larix gmelini*; TA *Tilia amurensis*; QM *Quercus mongolica*; UP *Ulmus pumila*).

According to the identification results based on the combination of the HSI+LiDAR, the optimal number of features selected in this study was 21, including 19 HSI features and 2 LiDAR features. The box plot (**Figures 9**, **10**) shows the capability of these 21 features in identifying 5 different tree species. For comparison purposes, the identification capability of the other three variables in the LiDAR features was also mapped. Although they were not selected for inclusion in the final fused data set, it can be seen from **Figure 10** that these three feature variables of LiDAR did have some effect on the identification of the tree species. For instance, ICA4 and MNF6 can well separate coniferous and broad-leaved trees, and the PCA5 and RGRI features differed obviously in *Juglans mandshurica* compared with other species. Similarly, PRI and MNF8 were beneficial for the identification of *Quercus mongolica*. Overall, the extracted features presented clear differences among the different tree species, and the differences between coniferous and broad-leaved trees were higher than that among different broad-leaved trees. In addition, as previously highlighted, the HSI features had a stronger capability to identify different tree species than the LiDAR features.

## 5 Discussion

In this study, identification of tree species using the HSI features alone could achieve a high level of accuracy, whereas the identification of tree species using LiDAR features on their own was less accurate. However, it was found that the combination of hyperspectral and LiDAR features could achieve an improvement in accuracy over HSI, and this finding is consistent with the conclusions of independent studies (Dalponte et al., 2012; Hartling et al., 2021). Zhao et al. (2020) has pointed out that the average spectrum of the tree canopy can represent the spectrum of individual trees and can weaken the influence of mixed pixels, making the approach more suitable for identification at the individual tree level than pixel-based identification. Most of the current research concerning the fusion of hyperspectral and LiDAR data for the identification of tree species is based on the use of airborne derived data. This type of study can cover large study areas, but the hyperspectral data typically has relatively low resolution. In our study, data acquisition was based on UAV hyperspectral images at low flight altitude, and the platform was capable of acquiring hyperspectral images with a spatial resolution of 10 cm. The hyperspectral data of individual trees obtained by a point cloud projection profile has more pixels, so that a large number of high quality pixels may be selected to generate the average spectral curve for the individual tree. Considering the complexity of natural mixed coniferous and broad-leaved forests, some mixed pixels do exist in the overlapping canopy boundaries. Therefore, the selection of sunlight pixels in the central area of the canopy to obtain the average spectral curve for individual trees can further reduce the influence of mixed pixels and improve the identification accuracy of tree species.

According to the results of tree species identification presented in **Figure 8**, and given the distinct differences of the preferred features in **Figures 9**, **10** among the five different tree species, it is clear that the features extracted and optimized are suitable for the identification of tree species investigated in the area under study. Using the feature selection capabilities of the RF algorithm, feature variables that have a positive impact on species identification can be selected, and the feature dimension can be greatly reduced without affecting the overall accuracy of identification, thereby reducing the complexity of species identification and improving the overall efficiency of the identification process.

The optimal feature combination includes 8 vegetation index features, 3 ICA transformed components, 5 MNF transformed components, 2 PCA transformed components, 1 original spectral band feature, and 2 LiDAR intensity features. Previous studies have also confirmed that the use of biochemical parameter-based vegetation indices as a means to characterize and identify tree species can effectively improve the accuracy of identification (Maschler et al., 2018; Shi et al., 2018b; Wu and Zhang, 2020). The present study further demonstrated that certain vegetation index highly correlated with biochemical parameters can be used for identification of tree species. Among them, the photochemical reflectance index (PRI) is very sensitive to the changes in the carotenoid content of vegetation, while the magnitudes of the anthocyanin reflectance index 1 (ARI1) and the anthocyanin reflectance index 2 (ARI2) values reflect mainly the contents of anthocyanin in leaf tissue. The red green ratio index (RGRI) is influenced mainly by both the anthocyanin and chlorophyll contents. However, Wu and Zhang (2020) and Maschler et al. (2018) pointed out that the identification of tree species based only on the use of several vegetation indices was not sufficiently robust, and further feature extraction methods should be included in the process. Therefore, in this study, the ICA, MNF, and PCA transformation methods were applied in the feature extraction process. Although it is not easy to explain these transformed components in the context of remote sensing mechanism, present research and other studies showed that ICA, PCA, and MNF transformations can compress useful high-dimensional hyperspectral data into useful components and improve the accuracy of identification. As shown in this study, ICA4 did achieve a good separation of coniferous and broad-leaved tree species. In general, the feature extraction capability of the ICA and MNF transformation methods, in the context of tree species identification, was superior to that of the PCA transformation method. It is worth noting that PCA, MNF and ICA transformations were used as methods for the reduction of data dimensions, and the useful information obtained from the dimensional reduction was concentrated in the front part of the components. For example, the higher the PCA component, the higher the amount of information; the higher the MNF components, the higher the signal-to-noise ratio. However, present results showed that the most “superior” component didn’t necessarily have the highest importance in identification of the tree species.

The two most important features of LiDAR were the mean of the first echo intensity and the mean of the total echo intensity, respectively. The first echo intensity can reduce multiple scattering effects in discrete echo systems. The subsequent LiDAR features included some features related to tree height such as the 95% quantile height of the first echo and the ratio of the number of point clouds at 80-100% height, these findings being similar to those reported previously (Korpela et al., 2010; Shi et al., 2018a). Although such studies and the present study have demonstrated that the features of tree height have a positive impact on the identification of tree species, it is believed that the tree height-related features will show some differences in identification capability for the different regions from the perspective of model applicability. Despite the fact that the spectrum of the same tree species in different regions may be affected by some factors (e.g., season, weather, sensors, etc.), this can be explained in terms of the remote sensing mechanism; also, the morphological structure of the same tree species does not vary significantly in normal forest stands, however, the tree height does vary greatly in different locations due to the growth cycle or environmental factors. Therefore, in the model transfer studies, it may be possible to give priority to the selection of spectral features, morphological structure of the tree and the echo intensity features in order to achieve enhanced results.

In general, most of the optimal features for identification of tree species were derived from hyperspectral data, while relatively few LiDAR features were utilized. The possible reasons include: (1) The LiDAR data contains less information than the hyperspectral data; (2) The high spatial resolution (10 cm) and high spectral resolution (2.1 nm) of the hyperspectral data obtained by the UAV system were of high quality, and were able to provide more accurate features for identification of tree species than airborne data; (3) The study area for this research was a natural mixed forest of coniferous and broadleaf trees with complex forest conditions and high canopy closure, which resulted in the incomplete acquisition of understory information by the LiDAR data, while the acquisition of the hyperspectral canopy information was almost unaffected. Therefore, the combined effect of these three factors contributed that the identification capability *via* the hyperspectral features was superior to that of LiDAR. Although the LiDAR data did not play such a prominent role as the hyperspectral data in the identification of the tree species, the LiDAR data were, nevertheless, indispensable to the success of this research. Due to the 3D information acquired by LiDAR, the data set has unparalleled advantages relative to other remote sensing in segmentation of individual trees. In this study, we used the distance-based point cloud clustering algorithm to segment the LiDAR data into individual trees basis and projected the profile for individual trees based on the point cloud for individual trees; the hyperspectral data was then segmented by the profile to obtain the hyperspectral data of individual trees. Therefore, the LiDAR and hyperspectral data complemented each other. The LiDAR data played an indispensable role in the acquisition of individual tree data, which laid the foundation for species identification at individual tree level. Thus, fusion of the HSI features and the LiDAR features achieved the best accuracy for identification of tree species.

It is noted that the combined UAV hyperspectral imagery and LiDAR data was used to identify tree species in natural mixed coniferous and broad-leaved forests in Northeast China. Although it proved possible to correctly classify the tree species, there is still a need for further in-depth and systematic studies. The availability of accurate data at the individual tree level is clearly the basis for ongoing research on tree species identification. In the future, we will explore whether HSI can be involved in individual tree segmentation and develop a point cloud optimization algorithm for individual tree segmentation to improve the effect of segmentation in natural coniferous and broad-leaved mixed forests. In the present work, the traditional machine learning algorithms were used for identification of tree species and feature selection. Future research can be conducted by using other exiting algorithms or new algorithms such as deep learning. Finally, due to the influences of temporal and spatial variations, the universality of achievements in remote sensing technology has always been a focus and difficulty for research. In this study, the best HSI and LiDAR features for identification of tree species in the study area were selected, however, their applicability to other geographical regions needs to be verified.

## 6 Conclusions

In this study, hyperspectral image and LiDAR data for a complex natural coniferous and broad-leaved mixed forest were performed by UAV. The accurate identification of tree species at the individual tree level was realized by combining the two types of data. The screening of optimal features for identification of tree species was conducted and a thematic map for tree species was created. By comparing the identification results for tree species with different data sources, it was demonstrated that the fusion of the hyperspectral and LiDAR data features resulted in improved accuracy for species identification. The optimal hyperspectral and LiDAR features for identification of tree species included the use of vegetation indices which were sensitive to chlorophyll, anthocyanin and carotene in the leaves, the partial components of the transformed ICA, MNF and PCA, and the LiDAR echo intensity features, respectively. The research will provide data support for diversity monitoring of forest species, forest biomass inversion and estimation of forest carbon stocks. The data can also act as a useful reference source for application of multi-source remote sensing technology in forestry.

## Data availability statement

The raw data supporting the conclusions of this article will be made available by the authors, without undue reservation.

## Author contributions

HZ carried out the experiments, performed data analysis, and drafted the manuscript. WL contributed to conception and design, supervised the research, and participated in manuscript drafting, revision and editing. HL, NM, KL, RC, TW, and ZR participated in part of the experimental investigations and data analysis. All authors contributed to the article and approved the submitted version.

## Funding

This article was supported by the Fundamental Research Funds for the Central Universities (2572021AW49), the National Natural Science Foundation of China (31971574) and the Joint Project of the Natural Science Foundation of Heilongjiang Province (LH2020C049).

## Conflict of interest

The authors declare that the research was conducted in the absence of any commercial or financial relationships that could be construed as a potential conflict of interest.

## Publisher’s note

All claims expressed in this article are solely those of the authors and do not necessarily represent those of their affiliated organizations, or those of the publisher, the editors and the reviewers. Any product that may be evaluated in this article, or claim that may be made by its manufacturer, is not guaranteed or endorsed by the publisher.
